# Community Exergaming and Maintenance-Related Outcomes in Older Adults: Multi-Wave Observational Evaluation

**DOI:** 10.2196/100007

**Published:** 2026-09-09

**Authors:** Yuezhong Liu, Bing Xun Chia, Herng Yeow Koh, Quoc Nam Tran Nguyen, Jiahui Jin, Kimberly Miracle Wei Yan Tan, Samuel Qing An Long, Yin-Leng Theng

**Affiliations:** 1 Ageing Research Institute for Society and Education, Nanyang Technological University, 61 Nanyang Drive, Academic Block North (ABN), #01A – 01, Singapore, Singapore, 637335, Singapore, 65 90679806; 2 Wee Kim Wee School of Communication and Information, Nanyang Technological University, Singapore, Singapore; 3 Sports Science & Management, National Institute of Education, Nanyang Technological University, Singapore, Singapore

**Keywords:** exergaming, older adults, digital health, physical activity, habit formation, motivation, implementation science, intergenerational, community-based intervention

## Abstract

**Background:**

Community exergaming may help older adults remain physically active by combining movement, enjoyment, and social interaction, but less is known about whether observed changes persist after supervised delivery ends in real-world community settings.

**Objective:**

This study evaluated immediate and maintenance-related outcomes of the International Singapore Intergenerational National Games, a community-delivered exergaming program for older adults in Singapore. We examined positive mental health, intrinsic motivation, habit strength, physical activity, and exploratory implementation determinants aligned with the capability, opportunity, and motivation–behavior (COM-B) framework.

**Methods:**

We conducted a multi-wave observational evaluation from October 2025 to January 2026 among 280 older adults recruited from 14 senior activity centers or active aging centers in Singapore. Assessments were conducted at baseline (T0), immediately after an 8-week supervised training phase (T1), after a 4-week unsupervised free-play phase (T2), and at a later follow-up for selected measures in 6 centers (T3). Immediate within-person changes were examined using 2-tailed paired *t* tests. Maintenance-related trajectories were examined using adjusted linear mixed-effects models. Exploratory analyses examined whether COM-B–aligned capability, opportunity, and motivation composites were associated with physical activity trajectories.

**Results:**

Participants had a mean age of 72.54 (SD 6.15) years, and 77.1% (216/280) were female. From T0 to T1, mean positive mental health increased from 55.19 (SD 9.79) to 57.59 (SD 8.70; Cohen *dz*=0.28; *P*<.001), mean intrinsic motivation increased from 5.94 (SD 1.00) to 6.29 (SD 0.81; Cohen *dz*=0.40; *P*<.001), and mean habit strength increased from 3.34 (SD 1.11) to 3.81 (SD 0.84; Cohen *dz*=0.42; *P*<.001). In adjusted mixed models, habit strength remained higher at T2 than at baseline (β=0.362, 95% CI .113-.611; *P*=.005), and intrinsic motivation remained higher at T2 (β=0.397, 95% CI .156-.638; *P*=.001) and T3 (β=0.305, 95% CI .009-.601; *P*=.04). Positive mental health was higher immediately after supervised training (β=4.140, 95% CI 2.169-6.111; *P*<.001) and at T2 (β=7.390, 95% CI 5.370-9.410; *P*<.001) than at baseline. Among the COM-B domains, only opportunity was associated with greater immediate posttraining physical activity improvement (time × opportunity interaction β=26.464, 95% CI 3.191-49.737; *P*=.03), with no evidence of a sustained interaction at T2.

**Conclusions:**

In this observational evaluation, positive mental health, intrinsic motivation, and habit strength were higher at T2 than at baseline among respondents with available follow-up data, and intrinsic motivation was also higher at the selected-center T3 follow-up. Because this study lacked a control group and attrition increased over time, these findings represent observed longitudinal trajectories rather than causal evidence of program effectiveness. Opportunity-related supports such as access, reminders, routines, staff facilitation, and peer participation may be useful when community exergaming transitions from supervised delivery to reduced-intensity free play, although this interpretation remains exploratory.

## Introduction

Community and group-based exergaming have attracted growing interest as a potentially scalable strategy for promoting later-life activity by combining movement, enjoyment, and social interaction. However, less is known about whether implementation-relevant gains persist once structured supervision is withdrawn in routine community settings. Across older adult studies, the most consistent benefits have been observed for balance and mobility, although effect sizes vary and intervention designs remain heterogeneous [1-5]. Cognitive and psychosocial outcomes are more mixed: reviews suggest possible benefits for mood, cognition, and health-related quality of life, but they also emphasize variability in delivery platforms, dose, comparator conditions, and measurement [2,6-8]. There is recent evidence likewise suggesting that different exergame emphases may yield different cognitive vs balance gains, underscoring the importance of intervention design [9].

Social and implementation questions remain especially important for community translation. Social outcomes are still measured inconsistently, yet there is available evidence suggesting that socially interactive or multiple-player exergaming formats may enhance loneliness-related and depressive outcomes more than solitary modes [10,11]. At the same time, follow-up beyond the immediate posttest remains limited, leaving uncertainty about what happens when staff prompting and structured supervision are reduced [2,5,12]. Qualitative and mixed methods studies further indicate that enjoyable gameplay alone is insufficient for implementation; familiarization, clear instructions, accessible design, confidence building, and social support all shape uptake and continued use [12-14].

The capability, opportunity, and motivation–behavior (COM-B) model provides a useful framework for examining these questions because it conceptualizes behavior as arising from capability, opportunity, and motivation [15]. Within this framework, intrinsic motivation and habit strength can be understood as motivational processes that may support behavioral maintenance, whereas opportunity reflects the social and environmental conditions that make continued activity possible after supervision is reduced. Self-determination theory further suggests that autonomy, competence, and relatedness-supportive delivery practices can foster more self-sustaining forms of motivation; recent consensus work has translated these principles into observable motivational behaviors that may be useful for community program delivery [16]. In a pragmatic community program, opportunity-related conditions such as access, reminders, peer presence, and light-touch support may be especially relevant once formal supervision decreases. The present study evaluated the International Singapore Intergenerational National Games (I-SING), a real-world community exergaming program delivered through senior activity centers and active aging centers in Singapore. The evaluation captured a supervised training phase, a deliberately designed 4-week unsupervised free-play phase, and later follow-up for selected measures.

Guided by the COM-B framework, we examined immediate and maintained changes in positive mental health, intrinsic motivation, habit strength, and physical activity together with descriptive feedback on program acceptability and barriers to continued participation. Because this study used an observational pretest-posttest design, the analyses were intended to describe trajectories and implementation-relevant associations rather than establish causal effectiveness.

This study tested 3 hypotheses:

- Hypothesis 1—positive mental health, intrinsic motivation, and habit strength would improve immediately after the supervised training phase.
- Hypothesis 2—intrinsic motivation and habit strength would remain higher during reduced-intensity follow-up than at baseline, whereas gains in positive mental health were expected to be less stable after structured social contact decreased.
- Hypothesis 3—higher COM-B–aligned capability, opportunity, and motivation scores would be associated with more favorable physical activity trajectories, with opportunity expected to be particularly relevant after supervision was reduced.

## Methods

### Study Design

**Figure 1.**
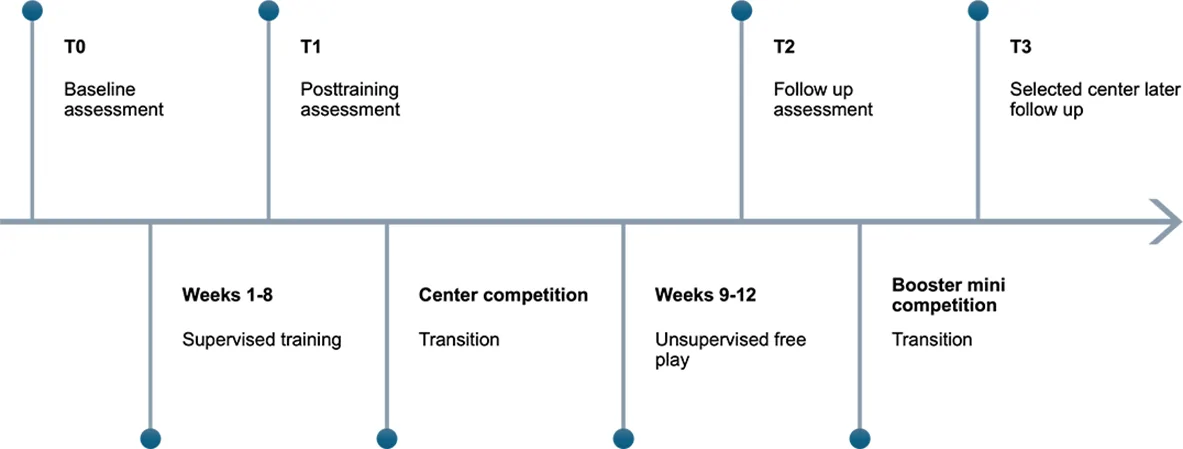
Study design and assessment timeline. T3 was conducted 1 week after the booster mini competition in 6 selected centers.

We conducted a multi-wave observational evaluation of the I-SING, a community-delivered intergenerational exergaming program for older adults in Singapore. Assessments were conducted before supervised training (T0), immediately after supervised training (T1), after a 4-week unsupervised free-play period (T2), and at a later follow-up for selected measures (T3). The design was pragmatic and did not include a control or comparison group. Therefore, this study could identify within-person changes and implementation-relevant associations, but it could not rule out secular trends, regression to the mean, center-level contextual effects, or other unmeasured confounders. The study design and assessment timeline are shown in Figure 1.

### Participants and Recruitment

Older adults were recruited from October 2025 to January 2026 through 14 participating senior activity centers and active aging centers using center-based outreach and posters. Eligibility followed the approved study protocol: participants were required to be 60 years of age or older, community ambulant, able to perform light physical activity, and free of a physical impairment that would preclude participation. Serious mental disorders were an exclusion criterion. Of 280 participants with baseline data, 2 (0.7%) withdrew at the program start, leaving 278 (99.3%) who entered the supervised phase. At the end of the 8-week supervised intervention, 243 participants completed the program; 242 (99.6%) provided analyzable posttraining (T1) survey data. After the 4-week unsupervised free-play phase, 230 participants contributed T2 data. Later follow-up (T3) was conducted only in 6 selected centers, where 120 participants were eligible for follow-up and 88 (73.3%) completed the T3 survey. Attrition analyses comparing baseline characteristics between completers and noncompleters at T1, T2, and T3 can be found in Tables S1 to S3 in Multimedia Appendix 1. At T1, completers were more likely than noncompleters to have previously played exergames and participated in I-SING. No clear baseline differences were observed between T2 completers and noncompleters. Among participants selected for T3 follow-up, completers differed from noncompleters in community center attendance frequency and were more likely to have previously played exergames and participated in I-SING. Other baseline demographic characteristics were generally comparable between completers and noncompleters across the assessment points.

### Program Procedures

The supervised phase consisted of 8 weekly sessions delivered over 8 weeks. After T1, a center competition was held, followed by a 4-week unsupervised free-play phase intended to observe naturalistic engagement under reduced staff prompting. A booster mini competition was introduced only after T2 data collection to reduce anticipation effects. Surveys required approximately 45 to 60 minutes at each wave.

### Outcome Measures

The focal outcomes for the present analyses were positive mental health, intrinsic motivation, and habit strength. Physical activity and COM-B–aligned implementation drivers were examined as exploratory, implementation-oriented outcomes. Detailed descriptions of additional measures collected for broader program evaluation, including healthy lifestyle, self-reported health, and health ownership, can be found in Multimedia Appendix 1.

### Ethical Considerations

This study was approved by the Nanyang Technological University Institutional Review Board (IRB-2023-321), including protocol amendments covering revised training formats and the unsupervised free-play period. All participants provided written informed consent before enrollment. Survey data were collected and analyzed in deidentified form to protect participant privacy and confidentiality. Participants received shopping vouchers as compensation for participation.

### Demographic Characteristics

A 9-item questionnaire assessed employment status, living arrangement, educational level, marital status, household size, perceived adequacy of money for daily expenses, frequency of community center visits, prior exergaming experience, and prior participation in I-SING. These variables were used to characterize the sample and, where relevant, as covariates.

### Positive Mental Health

Positive mental health was measured using the 14-item Mental Health Continuum–Short Form (MHC-SF), which assesses emotional, social, and psychological well-being [17]. Participants rated how often they had experienced each feeling during the previous month on a 6-point scale ranging from 0 (never) to 5 (every day). The 14 harmonized item scores were summed to produce total scores ranging from 0 to 70, with higher scores indicating better positive mental health.

### Motivation for Exergaming

Motivation for exergaming was assessed using an adapted version of the Motives for Physical Activity Measure–Revised (MPAM-R) [18]. Adaptation consisted of wording the original physical activity items to refer specifically to exergaming participation; no substantive changes were made to the response scale or intended construct. Items were rated on a 7-point scale ranging from 1 (not at all) to 7 (describes exactly). The present analyses focused on the intrinsic motivation score, with higher values indicating stronger intrinsically oriented motivation to engage in exergaming.

### Habit Strength

Habit strength was assessed using the 12-item Self-Report Habit Index (SRHI) [19]. Items were rated on a 5-point scale ranging from 1 (strongly disagree) to 5 (strongly agree). Item responses were averaged, with higher scores indicating stronger habit strength for the target behavior.

### Physical Activity

Physical activity was assessed using the Physical Activity Scale for the Elderly (PASE), a measure of leisure-time, household, and work-related activity in older adults [20]. Scores were calculated using the recommended weighted scoring procedure, with higher scores indicating greater physical activity. Because the PASE is a weighted composite of distinct physical activity domains rather than a unidimensional scale, internal consistency was not calculated.

### Program Experience and Feedback

At T3, participants from selected centers completed program feedback items on game preferences, enjoyment, recommendations, and suggestions for improvement. These responses were summarized descriptively to characterize program acceptability and practical implications for community delivery.

### Measurement Reliability

At T1, internal consistency was good for the 14-item MHC-SF (Cronbach α=0.89; n=242), the 14-item adapted MPAM-R intrinsic motivation score (Cronbach α=0.87; complete n=218), and the 12-item SRHI (Cronbach α=0.90; complete n=201). Designated missing value codes and values outside the prespecified response ranges were treated as missing before the coefficients were calculated. Internal consistency was not estimated for the PASE because its weighted total combines conceptually distinct activity domains rather than parallel items measuring a unidimensional construct.

### Operationalization of COM-B–Aligned Drivers

To explore implementation-relevant correlates of physical activity maintenance, we constructed 3 theory-informed composite indexes aligned with the COM-B domains of capability, opportunity, and motivation. These composites were pragmatic summary indicators for exploratory analysis and should not be interpreted as validated latent COM-B scales. Capability comprised health literacy, self-efficacy, information seeking, and health citizenship or control; opportunity comprised supportive accountability, perceived social support, responsibility and social contract, community actor, and collective efficacy; and motivation comprised healthy lifestyle, positive mental health, intrinsic and extrinsic motivation, habit strength, and self-reported health. Because the motivation composite included constructs that overlapped with the primary outcomes, we did not interpret this domain as an independent predictor of those same outcomes. Accordingly, the COM-B analyses were limited to physical activity trajectories and interpreted cautiously to minimize the risk of conceptual circularity.

Because this study focused on maintenance after supervised training, posttraining values were used preferentially to represent participants’ readiness for the reduced-intensity phase; baseline values were used only when posttraining values were unavailable. Component measures were standardized before aggregation, and higher composite scores indicated relatively greater capability, opportunity, or motivation. Full details on composite construction, weighting, equations, missing data handling, and a recommended sensitivity analysis excluding overlapping motivation indicators are provided in Multimedia Appendix 1.

### Statistical Analysis

Baseline characteristics were summarized using means and SDs for continuous variables and frequencies and percentages for categorical variables. The primary outcomes were positive mental health, intrinsic motivation, and habit strength. Immediate within-person change from T0 to T1 was examined using paired *t* tests based on complete pairs and reported as Cohen *dz* with 95% CIs. Maintenance across waves was examined using linear mixed-effects models with time as a categorical fixed effect and participant as a random intercept, adjusted for age, sex, and cohort and year. These covariates were selected a priori because age and sex are commonly associated with physical activity and psychosocial outcomes in older adults and cohort and year captured implementation differences across program cohorts. Analyses of PASE trajectories and COM-B driver interactions were prespecified as exploratory implementation-oriented analyses. All analyses used available data and were conducted in R (version 4.5.1; R Foundation for Statistical Computing). No imputation was performed; therefore, findings assume that missingness was adequately handled by the available-data mixed-model framework conditional on observed covariates and should be interpreted alongside attrition patterns.

## Results

### Sample and Baseline Characteristics

**Figure 2.**
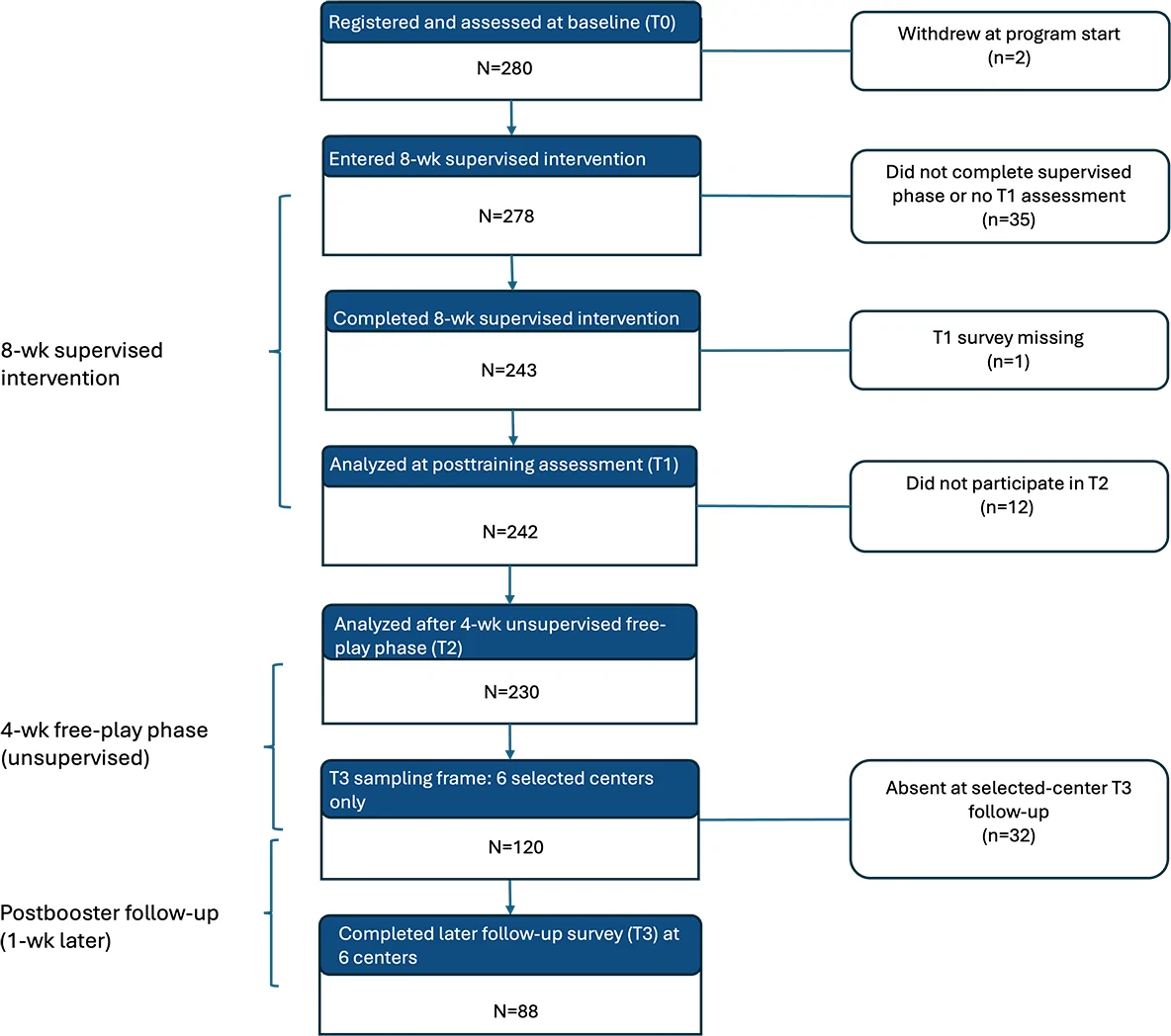
STROBE (Strengthening the Reporting of Observational Studies in Epidemiology)–style participant flow diagram. T0: baseline; T1: after an 8-week supervised training phase; T2: after a 4-week unsupervised free-play phase; T3: at a later follow-up for selected measures in 6 centers.

At baseline, the sample had a mean age of 72.54 (SD 6.15) years, 77.1% (216/280) of the participants were female, and 92.1% (258/280) identified as Chinese. Most participants were not working (244/277, 88.1%), and many reported frequent visits to community centers. Prior exergaming exposure was present but not dominant: 41.4% (115/278) of the participants had played exergames, and 21.9% (61/278) had participated in I-SING previously. Baseline sample characteristics are shown in Table 1. Participant flow is reported in accordance with STROBE (Strengthening the Reporting of Observational Studies in Epidemiology) guidance and shown in Figure 2.

**Table 1.** Participant demographics (baseline; N=280).

Characteristics	Values
Age (y), mean (SD)	72.54 (6.15)
Age group (y), n (%)	
60-64	22 (7.9)
65-69	72 (25.7)
70-74	84 (30)
75-79	64 (22.9)
≥80	38 (13.6)
Sex, n (%)	
Male	64 (22.9)
Female	216 (77.1)
Ethnicity, n (%)	
Chinese	258 (92.1)
Malay	10 (3.6)
Indian	8 (2.9)
Others	4 (1.4)
Spoken language, n (%)	
English	105 (37.5)
Mandarin	171 (61.1)
Other	4 (1.4)
Occupation status (n=277), n (%)	
Working	33 (11.9)
Not working	244 (88.1)
Living situation (n=278), n (%)	
Alone	84 (30.2)
With spouse	78 (28.1)
With spouse and children	56 (20.1)
With children	36 (12.9)
With friends or relatives	14 (5)
Tenant	1 (0.4)
Others	9 (3.2)
Highest level of education (n=278), n (%)	
No formal school education	38 (13.7)
Primary school	86 (30.9)
Secondary school	101 (36.3)
Diploma or polytechnic or preuniversity education	41 (14.7)
Degree, professional certifications, or higher	12 (4.3)
Marital status (n=278), n (%)	
Single, never married	33 (11.9)
Married	155 (55.8)
Cohabiting	1 (0.4)
Divorced	16 (5.8)
Separated	2 (0.7)
Widowed	71 (25.5)
Household members (n=278), n (%)	
Alone	71 (25.5)
2 people	93 (33.5)
3 people	46 (16.5)
4 people	29 (10.4)
5 people	14 (5)
More than 5 people	25 (9)
Perceived adequacy of daily expenses (n=278), n (%)	
Very insufficient	2 (0.7)
Not enough	15 (5.4)
Just enough	183 (65.8)
More than enough	71 (25.5)
Very abundant	7 (2.5)
Community center visits per week (n=278), n (%)	
1 time	22 (7.9)
2 times	34 (12.2)
3 times	50 (18)
4 times	59 (21.2)
5 times	72 (25.9)
More than 5 times	32 (11.5)
Others	9 (3.2)
Prior exergame experience (n=278), n (%)	
Played exergames before	115 (41.4)
Never played exergames before	163 (58.6)
Prior exergame experience (mo; n=90), mean (SD)	7.13 (13.65)
Prior participation in I-SING^a^ participants (n=278), n (%)	61 (21.9)
Previous I-SING program participated in (n=61), n (%)	
I-SING 2024—4 wk	2 (3.3)
I-SING 2024—8 wk	49 (80.3)
I-SING 2023—6 wk	0 (0)
I-SING 2023—12 wk	0 (0)
Others	10 (16.4)

a I-SING: International Singapore Intergenerational National Games.

### Immediate Program Effects

From T0 to T1, participants showed within-person increases in the 3 prespecified proximal outcomes. Mean positive mental health increased from 55.19 (SD 9.79) at T0 to 57.59 (SD 8.70) at T1 among 240 complete pairs (Cohen *dz*=0.279, 95% CI 0.149-0.407; *P*<.001). Mean intrinsic motivation increased from 5.94 (SD 1.00) to 6.29 (SD 0.81) among 166 paired observations (Cohen *dz*=0.398, 95% CI 0.239-0.555; *P*<.001). Mean habit strength increased from 3.34 (SD 1.11) to 3.81 (SD 0.84) among 143 paired observations (Cohen *dz*=0.423, 95% CI 0.252-0.594; *P*<.001). These results support hypothesis 1 but should be interpreted as changes observed after program participation rather than controlled intervention effects. Pair-specific sample sizes for the primary outcomes are reported in Table S4 in Multimedia Appendix 1.

Exploratory paired analyses of additional psychosocial variables are summarized in Table S5 in Multimedia Appendix 1. Briefly, small significant increases were observed for extrinsic motivation (*P*=.009) and social responsibility (*P*=.009), whereas collective efficacy decreased slightly (*P*=.03); no reliable immediate change was detected for self-efficacy (*P*=.24), supportive accountability (*P*=.31), perceived social support (*P*=.26), information seeking (*P*=.63), or health literacy (*P*=.73).

### Maintenance Under Reduced Intensity

Hypothesis 2 was partially supported. Habit strength was higher immediately after supervised training (β=0.291, 95% CI .047-.535; *P*=.02) and after the free-play phase at T2 (β=0.362, 95% CI .113-.611; *P*=.005) than at baseline. Intrinsic motivation was higher at T2 (β=0.397, 95% CI .156-.638; *P*=.001) and at the selected-center T3 follow-up (β=0.305, 95% CI .009-.601; *P*=.04) than at baseline. Contrary to the expectation that positive mental health gains would be less stable, positive mental health was higher both immediately after supervised training (β=4.140, 95% CI 2.169-6.111; *P*<.001) and at T2 (β=7.390, 95% CI 5.370-9.410; *P*<.001) than at baseline. The paired T0-to-T2 change in positive mental health was moderate (n=228; Cohen *dz*=0.520, 95% CI 0.382-0.658; *P*<.001).

These findings indicate that positive mental health, intrinsic motivation, and habit strength were all higher at T2 than at baseline among respondents with available follow-up data. Nevertheless, because this study lacked a control group and relied on available observations, the changes cannot be attributed causally to I-SING. Secular changes, contextual influences, and selective retention remain possible alternative explanations (Table 2).

**Table 2.** Primary and maintenance outcomes across study waves^a^.

Outcome and time point	Participants, n	Score, mean (SD)	Contrast vs baseline, β (95% CI)	*P* value	Paired effect size, Cohen *dz*
Habit strength (range 1-5)					
Before	278	3.319 (1.103)	Reference	—^b^	—
After	242	3.729 (0.836)	0.291 (0.047 to 0.535)	.02	0.423
T2^c^	230	3.729 (0.817)	0.362 (.113 to 0.611)	.005	0.382
Intrinsic motivation (range 1-7)					
Before	278	5.945 (0.982)	Reference	—	—
After	242	6.305 (0.764)	0.184 (–0.054 to 0.421)	.13	0.398
T2	230	6.403 (0.683)	0.397 (0.156 to 0.638)	.001	0.512
T3^d^	88	6.349 (0.988)	0.305 (0.009 to 0.601)	.04	0.291
Positive mental health (MHC-SF^e^ total; 0-70)					
Before	278	55.076 (9.726)	Reference	—	—
After	242	57.674 (8.712)	4.140 (2.169 to 6.111)	<.001	0.279
T2	230	60.622 (8.131)	7.390 (5.370 to 9.410)	<.001	0.520

a Mixed-effects models included time as a categorical fixed effect and participant as a random intercept and were adjusted for age, sex, and program cohort and year. Positive β values indicate scores higher than baseline. The n values represent the number of available observations at each wave. Paired effect sizes were calculated using complete pairs; pair-specific sample sizes are reported in Table S4 in Multimedia Appendix 1. For the Mental Health Continuum–Short Form, the T0-to-T1 effect size was based on 240 complete pairs, and the T0-to-T2 effect size was based on 228 complete pairs.

b Not applicable because baseline was the reference category; therefore, no P value or paired effect size was calculated.

c T2: after a 4-week unsupervised free-play phase.

d T3: at a later follow-up for selected measures in 6 centers.

e MHC-SF: Mental Health Continuum–Short Form.

Taken together, the adjusted models indicated that positive mental health, intrinsic motivation, and habit strength were higher at T2 than at baseline among respondents with available data. Intrinsic motivation was also higher at the selected-center T3 follow-up than at baseline. These results describe observed longitudinal trajectories and should not be interpreted as evidence that the reduced-intensity phase caused the outcomes to be maintained.

### COM-B Drivers and Physical Activity Maintenance Over Time

Hypothesis 3 examined whether higher COM-B–aligned capability, opportunity, and motivation were associated with differences in physical activity trajectories across baseline, immediately after training, and the T2 follow-up (Table 3). In mixed-effects models, only opportunity significantly moderated immediate change in physical activity after training (time × opportunity interaction β=26.464, 95% CI 3.191-49.737; *P*=.03). No significant time interactions were observed for capability or motivation, and no COM-B domain significantly moderated T2 physical activity change. In separate end point models, baseline PASE strongly predicted both posttraining and follow-up PASE scores, whereas opportunity showed only borderline evidence of association with posttraining PASE scores (detailed end point regression results can be found in Table S6 in Multimedia Appendix 1). Given the number of exploratory interactions tested, this result should be interpreted as a preliminary hypothesis-generating signal rather than definitive evidence that opportunity is more important than other COM-B domains.

**Table 3.** Associations between capability, opportunity, and motivation–behavior (COM-B) drivers and physical activity maintenance over time. Positive estimates indicate stronger increases in Physical Activity Scale for the Elderly among participants with higher standardized driver scores.

COM-B driver and interaction term	Regression coefficient, β (95% CI)	*P* value	Interpretation
Capability			
T1^a^ × capability	−9.332 (−32.331 to 13.666)	.42	No evidence of interaction
T2^b^ × capability	9.252 (–14.230 to 32.735)	.44	No evidence of interaction
Opportunity			
T1 × opportunity	26.464 (3.191 to 49.737)	.03	Higher opportunity associated with greater immediate increase in PASE^c^
T2 × opportunity	0.164 (–23.606 to 23.934)	.99	No evidence of interaction
Motivation			
T1 × motivation	10.015 (–10.159 to 30.190)	.33	No evidence of interaction
T2 × motivation	−6.623 (–27.145 to 13.899)	.52	No evidence of interaction

a T1: immediately after an 8-week supervised training phase.

b T2: after a 4-week unsupervised free-play phase.

c PASE: Positive estimates indicate greater increases in PASE among participants with higher standardized driver scores.

The COM-B domains were operationalized as follows: capability comprised health literacy, self-efficacy, information seeking, and control; opportunity comprised supportive accountability, perceived social support, responsibility and social contract, community actor, and collective efficacy; and motivation comprised healthy lifestyle, positive mental health, intrinsic and extrinsic motivation, habit, and self-reported health. The standardized COM-B driver scores were centered around 0, with means close to 0 and SDs ranging from 0.605 to 0.638, indicating comparable scaling across the 3 domains. These composites were exploratory and theory informed, and their results should not be interpreted as validated COM-B measurement scores.

Mixed-effects models assessed whether capability, opportunity, and motivation moderated physical activity trajectories from baseline to the posttraining and follow-up periods. Mean PASE scores increased modestly from baseline (mean 143.71, SD 66.42) to the posttraining time point (mean 147.81, SD 66.91) and declined at T2 (mean 137.02, SD 58.53). Among the 3 COM-B domains, only opportunity significantly moderated immediate posttraining change in physical activity. In separate end point models, baseline physical activity strongly predicted both posttraining and follow-up PASE scores, whereas opportunity showed only borderline evidence of association with posttraining PASE scores.

### Game Feedback From Selected Centers at T3

Because T3 feedback was available only for selected centers, these findings are presented descriptively. Overall, the exergaming component appeared to be well accepted. Apple Frenzy emerged as the most consistently preferred game and was commonly described as easy to play, enjoyable, relaxing, colorful, and easy to score. Fishing Frenzy was also frequently identified as a favorite because it was perceived as fun, active, and similar to exercise. Punch Punch and Whack-a-Mole were also popular, particularly among participants who valued excitement, reflex training, or challenges. In contrast, more cognitively demanding or faster-paced games such as Chinatown Rush and Red Light Rat Race appeared more polarizing. Participants also most often described Cat Stack, Don’t Forget to Wipe, and Ice Breaker negatively, typically because they were perceived as too difficult, tiring, frustrating, or hard to control. Detailed game-level results are provided in Table S7 in Multimedia Appendix 1.

Open-ended comments suggested that continued engagement during free play depended not only on game enjoyment but also on center-level opportunity conditions, including reminders, scheduling, staff support, and available space. This descriptive pattern was consistent with the quantitative finding that opportunity was the only COM-B domain associated with stronger immediate posttraining physical activity improvement.

Notably, participants often described Fishing Frenzy as enjoyable because it involved substantial movement and “helps me exercise.” This complements the supplementary functional movement mapping, within which Fishing Frenzy is characterized as a repeated sit-to-stand task with likely lower-limb strengthening and balance-related benefits. Thus, participant feedback supports the view that games with clear, functional, exercise-like movement demands may be especially acceptable in community exergaming for older adults.

## Discussion

### Principal Findings

This multi-wave observational evaluation examined whether changes in psychosocial and behavioral outcomes persisted after a community exergaming program transitioned from supervised training to reduced-intensity free play. Positive mental health, intrinsic motivation, and habit strength increased immediately after supervised training and were higher at T2 than at baseline among respondents with available data; intrinsic motivation was also higher at the selected-center T3 follow-up than at baseline. Opportunity was the only COM-B domain associated with greater immediate posttraining change in physical activity, and no COM-B domain moderated T2 change. These findings represent observed longitudinal trajectories and exploratory associations rather than causal program effects.

The immediate pattern is broadly consistent with earlier exergaming research. Reviews have repeatedly found the strongest and most consistent benefits in movement-related outcomes such as balance and mobility, whereas psychosocial effects are more heterogeneous across studies and measures [2,3,5,6]. The present study contributes something different: it evaluated a large, community-delivered program operating through routine service infrastructure in Singapore rather than a tightly controlled efficacy trial. This makes the findings especially relevant for implementation decisions in community aging services.

The maintenance-related findings extended beyond motivation and habit [13,14,18]. Positive mental health was also higher at T2 than at baseline, contrary to the expectation that mental health gains would be less stable after structured social contact decreased. The sustained differences in positive mental health, intrinsic motivation, and habit strength suggest that favorable outcomes were observed beyond the supervised phase among participants who remained in follow-up. However, attrition, the relatively short follow-up period, and the absence of direct free-play use data limit conclusions about maintenance in the full cohort and about whether these psychosocial and habit-related scores translated into continued exergaming participation.

Positive mental health was higher immediately after supervised training and at T2 than at baseline. This pattern suggests that the observed improvement was not confined to the supervised phase. However, because this study lacked a control group and the T2 analysis relied on available follow-up data, the higher score cannot be attributed causally to I-SING. Secular changes, center-level context, survey administration conditions, and selective retention may also have contributed to the observed trajectory.

The COM-B results provide a cautious implementation signal rather than a definitive mechanism test. Of the 3 COM-B domains, only opportunity was associated with stronger immediate posttraining physical activity change. In COM-B terms, opportunity refers to the environmental and social conditions that enable behavior, including access, social support, cues, and a facilitating context [15]. This interpretation is plausible in community exergaming because reminders, peer presence, accessible scheduling, and light-touch staff support may help older adults translate interest into actual participation. However, the opportunity result should be considered hypothesis generating because multiple interactions were tested, the composites were not validated COM-B scales, and center-level context may have contributed to the observed association.

The descriptive game feedback supports that same conclusion. Participants preferred games that combined intuitive movement, moderate pace, clear goals, and a rewarding sense of exercise. Fishing Frenzy and Apple Frenzy appeared to work well because they were both accessible and meaningfully active, with Fishing Frenzy often described as helping participants exercise. In contrast, games perceived as confusing, overly fast, poorly tracked, or physically unrewarding were less acceptable. Importantly, free-play barriers were often logistical rather than motivational, reinforcing the inference that access, reminders, scheduling, and space matter as much as game enjoyment once supervision recedes [13,15].

Several limitations should be acknowledged. First, the observational pretest-posttest design lacked a control group, limiting causal inference and preventing attribution of the observed changes to I-SING alone. Second, analyses used available observations, and attrition reduced the sample for paired comparisons and later follow-up. Selective retention of participants who were more engaged, healthier, or more familiar with exergaming may have contributed to the apparent maintenance-related trajectories. Third, T3 was restricted to selected centers, limiting the generalizability of those findings. Fourth, no automated logs or equivalent individual-level records of free-play exposure were available; consequently, higher motivation and habit scores cannot be assumed to represent continued exergaming participation. Fifth, the PASE was self-reported. Sixth, the COM-B composites were theory-informed pragmatic indexes rather than validated measures, and the motivation composite contained constructs that overlapped conceptually with the primary outcomes. Finally, no dedicated validated social outcome battery was administered, so conclusions regarding loneliness, social connectedness, or social participation cannot be drawn. Future studies should include controlled comparisons, direct measures of program exposure and adherence, validated social health outcomes, and sensitivity analyses addressing missing data and center-level clustering.

### Conclusions

In this multi-wave observational evaluation of community exergaming for older adults, positive mental health, intrinsic motivation, and habit strength were higher at T2 than at baseline among respondents with available follow-up data, and intrinsic motivation was also higher at the selected-center T3 follow-up. These findings suggest maintenance-related signals across both psychosocial and behavioral outcomes, but the uncontrolled design, attrition, selected-center follow-up, and absence of direct free-play exposure data preclude causal conclusions. Opportunity-related supports such as access, reminders, scheduling, staff facilitation, and peer participation may be useful when programs transition from supervised sessions to reduced-intensity free play, although this interpretation remains exploratory. Future controlled studies with consistent cross-wave scoring, validated social outcomes, and direct tracking of program exposure are needed to clarify maintenance pathways.

## Supplementary material

10.2196/100007 Multimedia Appendix 1 Supplementary methods and results.

## Abbreviations

COM-B: capability, opportunity, and motivation–behavior
I-SING: International Singapore Intergenerational National Games
MHC-SF: Mental Health Continuum–Short Form
MPAM-R: Motives for Physical Activity Measure–Revised
PASE: Physical Activity Scale for the Elderly
SRHI: Self-Report Habit Index
STROBE: Strengthening the Reporting of Observational Studies in Epidemiology
