# Supplementary material for: Community Exergaming and Maintenance-Related Outcomes in Older Adults: Multi-Wave Observational Evaluation
Source: JMIR Aging. 2026 Sep 9;9:e100007. doi: 10.2196/100007 (PMC13557100; doi:10.2196/100007)
Supplement: Supplementary file 1 [file aging-v9-e100007-s001.docx]

**Community Exergaming and Maintenance-Related Outcomes in Older Adults in Singapore: Multi-wave Observational Evaluation**

**Authors:** Yuezhong Liu^1*^, Bing Xun Chia^1^, Herng Yeow Koh^1^, Quoc Nam Tran Nguyen^1^, Jiahui Jin^2^, Kimberly Miracle Wei Yan Tan^1^, Samuel Qing An Long^3^, Yin-Leng Theng^1,2^.

^1^Ageing Research Institute for Society and Education (ARISE), Nanyang Technological University, Singapore;

^2^Wee Kim Wee School of Communication and Information (WKWSCI), Nanyang Technological University, Singapore;

^3^Physical Education & Sports Science, National Institute of Education (NIE), Nanyang Technological University (NTU), Singapore;

*Corresponding Author: Yuezhong Liu, Ageing Research Institute for Society and Education (ARISE), Nanyang Technological University, 61 Nanyang Drive, Academic Block North (ABN), #01A – 01, Singapore. 637335. Tel: +65 9067 9806. Fax: +65 6513 2140. Email: yz.liu@ntu.edu.sg

**Supplementary Methods and Results**

***Operationalisation of COM-B–Aligned Drivers***

To examine implementation-relevant behavioural drivers associated with maintenance outcomes, we operationalised Capability, Opportunity, and Motivation as composite indices derived from conceptually aligned psychosocial measures. This approach was guided by the COM-B framework, which proposes that behaviour is shaped by individuals’ capability to perform it, the opportunities afforded by their social and environmental context, and the motivational processes that energise and sustain it. In the present study, the composite approach was used to reduce model complexity, improve interpretability, and enable theory-informed testing of whether broader COM-B–aligned determinants were associated with maintenance-related outcomes over time.

***Conceptual Mapping of Measures to COM-B Domains***

Measures were assigned to COM-B domains on theoretical grounds. Capability was defined as the set of personal resources relevant to understanding, managing, and carrying out health-related behaviour. It comprised health literacy, self-efficacy, information-seeking behaviour, and health citizenship/control. These measures were selected because they reflect participants’ perceived knowledge, confidence, self-regulatory capacity, and sense of personal agency in relation to health behaviour.

Opportunity was defined as the interpersonal and contextual conditions that could facilitate or constrain sustained engagement. It comprised supportive accountability, perceived social support, responsibility/social contract, community actor, and collective efficacy. These constructs were included because they capture the extent to which participants’ social environment, community context, and relational expectations may support or reinforce ongoing activity and programme engagement.

Motivation was defined as the affective, cognitive, and behavioural processes that direct and sustain health behaviour. It comprised healthy lifestyle, positive mental health and well-being, intrinsic and extrinsic motivation, habit strength, and self-reported health. These measures were included because they reflect the degree to which participants are psychologically engaged, behaviourally oriented, and personally inclined toward sustaining activity and health-promoting behaviour over time.

**Timing of COM-B Composite Construction**

Because the primary interest of the study was maintenance after the supervised phase of the intervention, COM-B driver scores were constructed to reflect participants’ behavioural resources and implementation conditions during or immediately after supervised training. Accordingly, post-training values were used preferentially when available. This timing decision was made to better capture the state participants carried into the reduced-intensity or follow-up phase, rather than relying solely on baseline characteristics that may no longer reflect their post-intervention readiness for maintenance.

Where post-training values for a component measure were unavailable, the corresponding baseline value was used as a fallback indicator to minimise unnecessary case loss and preserve the analytic sample. Thus, the resulting COM-B composites should be interpreted as representing participants’ most proximal observed capability, opportunity, and motivation entering the maintenance phase.

**Standardisation and Composite Score Construction**

Because the component measures were drawn from different instruments and used different response scales, each component was first standardised to a common metric prior to aggregation. Specifically, variables were transformed into z-scores using the sample mean and standard deviation as shown in Equation 1. Standardisation ensured that no single component exerted undue influence on the composite solely because of its original scale range.

$$\begin{aligned} z=\frac{x-\overline{x}}{sd} \#\left( 1 \right) \end{aligned}$$

Following standardisation, each COM-B domain score was calculated as the mean of the standardised component scores within that domain. Components were weighted equally so that each construct contributed comparably to the final composite. Thus, higher composite scores indicated relatively greater levels of the corresponding COM-B domain within the sample.

For participant *i*, the Capability composite was defined as the mean of the standardised values of health literacy (HL), self-efficacy (SE), information-seeking behaviour (ISB), and health citizenship/control (HCON) as shown in Equation 2. The Opportunity composite was defined as the mean of the standardised values of health climate / supportive accountability (HCSA), perceived social support (PSS), social responsibility (SS), community actor (CA), and collective efficacy (CE) as shown in Equation 3. The Motivation composite was defined as the mean of the standardised values of healthy lifestyle (HLS), positive mental health (MHC-SF), intrinsic motivation (IM), extrinsic motivation (EM), habit strength (HAB), and self-reported health indicators (SRH) as shown in Equation 4.

$\begin{aligned} {Capability}_{i}=\frac{z\left( {HL}_{i} \right)+z\left( {SE}_{i} \right)+z\left( {ISB}_{i} \right)+z\left( {HCONntrol}_{i} \right)}{4}\#\left( 2 \right) \end{aligned}$

$\begin{aligned} {Opportunity}_{i}=\frac{z\left( {HCSA}_{i} \right)+z\left( {PSS}_{i} \right)+z\left( {SS}_{i} \right)+z\left( {CA}_{i} \right)+z\left( {CE}_{i} \right)}{5}\#\left( 3 \right) \end{aligned}$

$\begin{aligned} {Motivation}_{i}=\frac{z\left( {HLS}_{i} \right)+z\left( {MHC}_{i} \right)+z\left( {IM}_{i} \right)+z\left( {EM}_{i} \right)+z\left( {HAB}_{i} \right)+z\left( {SRH}_{i} \right)}{k}\#\left( 4 \right) \end{aligned}$

where *k* is the number of non-missing standardised motivation indicators included for that participant.

**Handling of Missing Data in Composite Construction**

COM-B domain scores were computed using all available standardised components within each domain. Thus, when one or more component measures were missing, the composite was calculated as the mean of the non-missing components rather than being treated as entirely missing. This approach allowed participants with partial data to contribute to the analysis while maintaining the domain-based structure of the COM-B framework. Higher composite scores, therefore, reflected higher relative levels of the available indicators contributing to that domain.

**Rationale for Equal Weighting**

All component measures were weighted equally within each COM-B domain. Equal weighting was chosen because the present study was theory-driven and exploratory with respect to the integration of multiple psychosocial constructs into broader implementation-relevant COM-B drivers. In the absence of strong a priori or empirical justification for differential weighting, equal weighting provided a transparent and replicable method of composite construction while avoiding overinterpretation of the relative importance of individual indicators.

**Analytic Use of COM-B Composites**

The COM-B composites were used as explanatory variables in longitudinal analyses of maintenance-related outcomes. In particular, the Capability, Opportunity, and Motivation composites were entered into models examining whether participants with higher COM-B–aligned driver scores demonstrated stronger maintenance trajectories over time. Positive associations or positive time-by-COM-B interaction effects were interpreted as indicating that relatively greater perceived capability, opportunity, or motivation was associated with more favourable maintenance patterns after supervised training had ended.

**2. Supplementary Outcome Measures:**

The focal outcomes for the present analyses were positive mental health, intrinsic motivation, and habit strength. Physical activity and COM-B–aligned implementation drivers were examined as exploratory, implementation-oriented outcomes.

***Healthy lifestyle.***

Healthy lifestyle was assessed using an adapted 5-item composite based on Adams et al. (2016), covering smoking, leisure-time exercise, fruit and vegetable intake, binge/heavy drinking, and sleep duration. Positive behaviours were defined as not smoking, engaging in leisure-time exercise, consuming at least two servings each of fruits and vegetables daily, not engaging in binge/heavy drinking, and obtaining at least 7 hours of sleep per night. Smoking and binge/heavy drinking were reverse-coded. The measure was analysed as a count of healthy behaviours and, where relevant, as a dichotomous indicator of meeting all five criteria. For descriptive purposes, participants were classified as low (0-1), medium (2-3), or high (4-5) on healthy lifestyle.

***Self-reported physical and mental health.***

Self-reported health was assessed using the 12-Item Short-Form Health Survey (SF-12) (Ware et al., 1996). Standard scoring procedures were used to derive the Physical Component Summary (PCS-12) and Mental Component Summary (MCS-12), with higher scores indicating better perceived health.

***Health ownership.***

Health ownership was assessed using a self-developed exploratory measure informed by the concept of taking control of, accepting responsibility for, and being accountable for one’s health-related outcomes (Tlusty & Hanna, 2021). The measure included domains reflecting health climate, health citizenship, health contract/responsibility, and health literacy. Because this was an adapted exploratory measure, its internal consistency and dimensional structure were evaluated in the present sample before substantive analyses.

**3. Supplementary Results:**

## Attrition analyses compared baseline characteristics between completers and non-completers at T1 and T2 using the full baseline cohort (N=280). The T3 analysis was restricted to the 120 participants selected for follow-up, to distinguish follow-up nonresponse from selection into the T3 subsample.

## Table S1: Baseline characteristics of T1 (n = 280) completers (n = 242) and non-completers (n =38)

| **Characteristic** | **Category** | **Completers, n=242** | **Non-completers, n=38** | **P value** |
| --- | --- | --- | --- | --- |
| **Age (years)** |  | 72.52 (6.31) | 72.71 (5.10) | .834 |
| **Age band** |  |  |  | .347 |
|  | 60-64 | 21 (8.7%) | 1 (2.6%) |  |
|  | 65-69 | 60 (24.8%) | 12 (31.6%) |  |
|  | 70-74 | 75 (31.0%) | 9 (23.7%) |  |
|  | 75-79 | 52 (21.5%) | 12 (31.6%) |  |
|  | 80+ | 34 (14.0%) | 4 (10.5%) |  |
| **Gender** |  |  |  | .839 |
|  | Male | 55 (22.7%) | 9 (23.7%) |  |
|  | Female | 187 (77.3%) | 29 (76.3%) |  |
| **Ethnicity** |  |  |  | .107 |
|  | Chinese | 223 (92.1%) | 35 (92.1%) |  |
|  | Malay | 10 (4.1%) | 0 (0.0%) |  |
|  | Indian | 5 (2.1%) | 3 (7.9%) |  |
|  | Others | 4 (1.7%) | 0 (0.0%) |  |
| **Spoken language** |  |  |  | .319 |
|  | English | 87 (36.0%) | 18 (47.4%) |  |
|  | Mandarin | 151 (62.4%) | 20 (52.6%) |  |
|  | Other | 4 (1.7%) | 0 (0.0%) |  |
| **Occupation status** |  |  |  | .183 |
|  | Working | 26 (10.9%) | 7 (18.4%) |  |
|  | Not working | 213 (89.1%) | 31 (81.6%) |  |
|  | Missing | 3 | 0 |  |
| **Living situation** |  |  |  | .084 |
|  | Alone | 75 (31.2%) | 9 (23.7%) |  |
|  | With spouse | 72 (30.0%) | 6 (15.8%) |  |
|  | With spouse and children | 45 (18.8%) | 11 (28.9%) |  |
|  | With children | 31 (12.9%) | 5 (13.2%) |  |
|  | With friends/relatives | 9 (3.8%) | 5 (13.2%) |  |
|  | Tenant | 1 (0.4%) | 0 (0.0%) |  |
|  | Others | 7 (2.9%) | 2 (5.3%) |  |
|  | Missing | 2 | 0 |  |
| **Highest level of education** |  |  |  | .759 |
|  | No formal school education | 34 (14.2%) | 4 (10.5%) |  |
|  | Primary school | 75 (31.2%) | 11 (28.9%) |  |
|  | Secondary school | 86 (35.8%) | 15 (39.5%) |  |
|  | Diploma / Polytechnic / Pre-university | 36 (15.0%) | 5 (13.2%) |  |
|  | Degree / Professional Certifications and above | 9 (3.8%) | 3 (7.9%) |  |
|  | Missing | 2 | 0 |  |
| **Marital status** |  |  |  | .376 |
|  | Single, never married | 30 (12.5%) | 3 (7.9%) |  |
|  | Married | 135 (56.2%) | 20 (52.6%) |  |
|  | Cohabiting | 1 (0.4%) | 0 (0.0%) |  |
|  | Divorced | 11 (4.6%) | 5 (13.2%) |  |
|  | Separated | 2 (0.8%) | 0 (0.0%) |  |
|  | Widowed | 61 (25.4%) | 10 (26.3%) |  |
|  | Missing | 2 | 0 |  |
| **Household members** |  |  |  | .095 |
|  | Alone | 63 (26.2%) | 8 (21.1%) |  |
|  | 2 people | 81 (33.8%) | 12 (31.6%) |  |
|  | 3 people | 38 (15.8%) | 8 (21.1%) |  |
|  | 4 people | 28 (11.7%) | 1 (2.6%) |  |
|  | 5 people | 9 (3.8%) | 5 (13.2%) |  |
|  | More than 5 people | 21 (8.8%) | 4 (10.5%) |  |
|  | Missing | 2 | 0 |  |
| **Perceived adequacy of daily expenses** |  |  |  | .792 |
|  | Very insufficient | 2 (0.8%) | 0 (0.0%) |  |
|  | Not enough | 13 (5.4%) | 2 (5.3%) |  |
|  | Just enough | 159 (66.2%) | 24 (63.2%) |  |
|  | More than enough | 61 (25.4%) | 10 (26.3%) |  |
|  | Very abundant | 5 (2.1%) | 2 (5.3%) |  |
|  | Missing | 2 | 0 |  |
| **Community centre visits per week** |  |  |  | .675 |
|  | 1 time | 17 (7.1%) | 5 (13.2%) |  |
|  | 2 times | 27 (11.2%) | 7 (18.4%) |  |
|  | 3 times | 43 (17.9%) | 7 (18.4%) |  |
|  | 4 times | 53 (22.1%) | 6 (15.8%) |  |
|  | 5 times | 64 (26.7%) | 8 (21.1%) |  |
|  | More than 5 times | 28 (11.7%) | 4 (10.5%) |  |
|  | Others | 8 (3.3%) | 1 (2.6%) |  |
|  | Missing | 2 | 0 |  |
| **Played exergames before** |  |  |  | .007 |
|  | Yes | 107 (44.6%) | 8 (21.1%) |  |
|  | No | 133 (55.4%) | 30 (78.9%) |  |
|  | Missing | 2 | 0 |  |
| **Months of prior exergame experience** |  | 7.30 (14.03) | 5.14 (8.36) | .555 |
|  | Missing | 159 | 31 |  |
| **Participated in I-SING previously** |  |  |  | .006 |
|  | Yes | 59 (24.6%) | 2 (5.3%) |  |
|  | No | 181 (75.4%) | 36 (94.7%) |  |
|  | Missing | 2 | 0 |  |
| **Programme cohort** |  |  |  | .776 |
|  | I-SING2024 (4 week) | 2 (3.4%) | 0 (0.0%) |  |
|  | I-SING2024 (8 week) | 47 (79.7%) | 2 (100.0%) |  |
|  | I-SING2023 (6 week) | 0 (0.0%) | 0 (0.0%) |  |
|  | I-SING2023 (12 week) | 0 (0.0%) | 0 (0.0%) |  |
|  | Others | 10 (16.9%) | 0 (0.0%) |  |
|  | Missing | 183 | 36 |  |

## *Note*. Values are mean (SD) or n (%). Percentages are based on nonmissing observations within each completion group. P values are unadjusted. Continuous variables were compared using Welch two-sample t tests; binary categorical variables using Fisher exact tests; and multilevel categorical variables using Monte Carlo permutation Pearson chi-square tests (20,000 replicates).

## Table S2: Baseline characteristics of T2 completers and non-completers

| **Characteristic** | **Category** | **Completers, n=230** | **Non-completers, n=50** | **P value** |
| --- | --- | --- | --- | --- |
| **Age (years)** |  | 72.30 (6.12) | 73.66 (6.23) | .165 |
| **Age group** |  |  |  | .482 |
|  | 60-64 | 18 (7.8%) | 4 (8.0%) |  |
|  | 65-69 | 64 (27.8%) | 8 (16.0%) |  |
|  | 70-74 | 67 (29.1%) | 17 (34.0%) |  |
|  | 75-79 | 52 (22.6%) | 12 (24.0%) |  |
|  | 80+ | 29 (12.6%) | 9 (18.0%) |  |
| **Gender** |  |  |  | .853 |
|  | Male | 52 (22.6%) | 12 (24.0%) |  |
|  | Female | 178 (77.4%) | 38 (76.0%) |  |
| **Ethnicity** |  |  |  | .535 |
|  | Chinese | 213 (92.6%) | 45 (90.0%) |  |
|  | Malay | 7 (3.0%) | 3 (6.0%) |  |
|  | Indian | 6 (2.6%) | 2 (4.0%) |  |
|  | Others | 4 (1.7%) | 0 (0.0%) |  |
| **Spoken language** |  |  |  | .162 |
|  | English | 84 (36.5%) | 21 (42.0%) |  |
|  | Mandarin | 144 (62.6%) | 27 (54.0%) |  |
|  | Other | 2 (0.9%) | 2 (4.0%) |  |
| **Occupation status** |  |  |  | 1.000 |
|  | Working | 27 (11.9%) | 6 (12.0%) |  |
|  | Not working | 200 (88.1%) | 44 (88.0%) |  |
|  | Missing | 3 | 0 |  |
| **Living situation** |  |  |  | .758 |
|  | Alone | 71 (31.1%) | 13 (26.0%) |  |
|  | With spouse | 66 (28.9%) | 12 (24.0%) |  |
|  | With spouse and children | 44 (19.3%) | 12 (24.0%) |  |
|  | With children | 28 (12.3%) | 8 (16.0%) |  |
|  | With friends/relatives | 12 (5.3%) | 2 (4.0%) |  |
|  | Tenant | 1 (0.4%) | 0 (0.0%) |  |
|  | Others | 6 (2.6%) | 3 (6.0%) |  |
|  | Missing | 2 | 0 |  |
| **Highest level of education** |  |  |  | .441 |
|  | No formal school education | 28 (12.3%) | 10 (20.0%) |  |
|  | Primary school | 71 (31.1%) | 15 (30.0%) |  |
|  | Secondary school | 82 (36.0%) | 19 (38.0%) |  |
|  | Diploma / Polytechnic / Pre-university | 37 (16.2%) | 4 (8.0%) |  |
|  | Degree / Professional Certifications and above | 10 (4.4%) | 2 (4.0%) |  |
|  | Missing | 2 | 0 |  |
| **Marital status** |  |  |  | .730 |
|  | Single, never married | 28 (12.3%) | 5 (10.0%) |  |
|  | Married | 127 (55.7%) | 28 (56.0%) |  |
|  | Cohabiting | 1 (0.4%) | 0 (0.0%) |  |
|  | Divorced | 11 (4.8%) | 5 (10.0%) |  |
|  | Separated | 2 (0.9%) | 0 (0.0%) |  |
|  | Widowed | 59 (25.9%) | 12 (24.0%) |  |
|  | Missing | 2 | 0 |  |
| **Household members** |  |  |  | .816 |
|  | Alone | 58 (25.4%) | 13 (26.0%) |  |
|  | 2 people | 75 (32.9%) | 18 (36.0%) |  |
|  | 3 people | 39 (17.1%) | 7 (14.0%) |  |
|  | 4 people | 26 (11.4%) | 3 (6.0%) |  |
|  | 5 people | 11 (4.8%) | 3 (6.0%) |  |
|  | More than 5 people | 19 (8.3%) | 6 (12.0%) |  |
|  | Missing | 2 | 0 |  |
| **Perceived adequacy of daily expenses** |  |  |  | .557 |
|  | Very insufficient | 1 (0.4%) | 1 (2.0%) |  |
|  | Not enough | 11 (4.8%) | 4 (8.0%) |  |
|  | Just enough | 153 (67.1%) | 30 (60.0%) |  |
|  | More than enough | 58 (25.4%) | 13 (26.0%) |  |
|  | Very abundant | 5 (2.2%) | 2 (4.0%) |  |
|  | Missing | 2 | 0 |  |
| **Community centre visits per week** |  |  |  | .277 |
|  | 1 time | 15 (6.6%) | 7 (14.0%) |  |
|  | 2 times | 25 (11.0%) | 9 (18.0%) |  |
|  | 3 times | 42 (18.4%) | 8 (16.0%) |  |
|  | 4 times | 49 (21.5%) | 10 (20.0%) |  |
|  | 5 times | 62 (27.2%) | 10 (20.0%) |  |
|  | More than 5 times | 26 (11.4%) | 6 (12.0%) |  |
|  | Others | 9 (3.9%) | 0 (0.0%) |  |
|  | Missing | 2 | 0 |  |
| **Played exergames before** |  |  |  | .155 |
|  | Yes | 99 (43.4%) | 16 (32.0%) |  |
|  | No | 129 (56.6%) | 34 (68.0%) |  |
|  | Missing | 2 | 0 |  |
| **Months of prior exergame experience** |  | 7.58 (14.42) | 4.17 (6.42) | .176 |
|  | Missing | 152 | 38 |  |
| **Participated in I-SING previously** |  |  |  | .062 |
|  | Yes | 55 (24.1%) | 6 (12.0%) |  |
|  | No | 173 (75.9%) | 44 (88.0%) |  |
|  | Missing | 2 | 0 |  |
| **Programme cohort** |  |  |  | .443 |
|  | I-SING2024 (4 week) | 2 (3.6%) | 0 (0.0%) |  |
|  | I-SING2024 (8 week) | 43 (78.2%) | 6 (100.0%) |  |
|  | I-SING2023 (6 week) | 0 (0.0%) | 0 (0.0%) |  |
|  | I-SING2023 (12 week) | 0 (0.0%) | 0 (0.0%) |  |
|  | Others | 10 (18.2%) | 0 (0.0%) |  |
|  | Missing | 175 | 44 |  |

## *Note*. Values are mean (SD) or n (%). Percentages are based on nonmissing observations within each completion group. P values are unadjusted. Continuous variables were compared using Welch two-sample t tests; binary categorical variables using Fisher exact tests; and multilevel categorical variables using Monte Carlo permutation Pearson chi-square tests (20,000 replicates).

## Table S3: Baseline characteristics of T3 completers and non-completers

| **Characteristic** | **Category** | **Completers, n=88** | **Non-completers, n=32** | **P value** |
| --- | --- | --- | --- | --- |
| **Age (years)** |  | 71.44 (6.21) | 72.88 (5.48) | .227 |
| **Age group** |  |  |  | .452 |
|  | 60-64 | 10 (11.4%) | 2 (6.2%) |  |
|  | 65-69 | 25 (28.4%) | 7 (21.9%) |  |
|  | 70-74 | 27 (30.7%) | 10 (31.2%) |  |
|  | 75-79 | 17 (19.3%) | 11 (34.4%) |  |
|  | 80+ | 9 (10.2%) | 2 (6.2%) |  |
| **Gender** |  |  |  | .792 |
|  | Male | 17 (19.3%) | 5 (15.6%) |  |
|  | Female | 71 (80.7%) | 27 (84.4%) |  |
| **Ethnicity** |  |  |  | .535 |
|  | Chinese | 82 (93.2%) | 28 (87.5%) |  |
|  | Malay | 1 (1.1%) | 0 (0.0%) |  |
|  | Indian | 3 (3.4%) | 3 (9.4%) |  |
|  | Others | 2 (2.3%) | 1 (3.1%) |  |
| **Spoken language** |  |  |  | .141 |
|  | English | 32 (36.4%) | 17 (53.1%) |  |
|  | Mandarin | 56 (63.6%) | 15 (46.9%) |  |
|  | Other | 0 (0.0%) | 0 (0.0%) |  |
| **Occupation status** |  |  |  | .081 |
|  | Working | 6 (6.8%) | 6 (18.8%) |  |
|  | Not working | 82 (93.2%) | 26 (81.2%) |  |
| **Living situation** |  |  |  | .769 |
|  | Alone | 32 (36.4%) | 7 (21.9%) |  |
|  | With spouse | 23 (26.1%) | 11 (34.4%) |  |
|  | With spouse and children | 18 (20.5%) | 7 (21.9%) |  |
|  | With children | 8 (9.1%) | 4 (12.5%) |  |
|  | With friends/relatives | 5 (5.7%) | 2 (6.2%) |  |
|  | Tenant | 1 (1.1%) | 0 (0.0%) |  |
|  | Others | 1 (1.1%) | 1 (3.1%) |  |
| **Highest level of education** |  |  |  | .184 |
|  | No formal school education | 9 (10.2%) | 3 (9.4%) |  |
|  | Primary school | 31 (35.2%) | 8 (25.0%) |  |
|  | Secondary school | 36 (40.9%) | 12 (37.5%) |  |
|  | Diploma / Polytechnic / Pre-university | 9 (10.2%) | 4 (12.5%) |  |
|  | Degree / Professional Certifications and above | 3 (3.4%) | 5 (15.6%) |  |
| **Marital status** |  |  |  | .969 |
|  | Single, never married | 12 (13.6%) | 4 (12.5%) |  |
|  | Married | 48 (54.5%) | 19 (59.4%) |  |
|  | Cohabiting | 0 (0.0%) | 0 (0.0%) |  |
|  | Divorced | 7 (8.0%) | 2 (6.2%) |  |
|  | Separated | 0 (0.0%) | 0 (0.0%) |  |
|  | Widowed | 21 (23.9%) | 7 (21.9%) |  |
| **Household members** |  |  |  | .286 |
|  | Alone | 25 (28.4%) | 7 (21.9%) |  |
|  | 2 people | 21 (23.9%) | 12 (37.5%) |  |
|  | 3 people | 16 (18.2%) | 4 (12.5%) |  |
|  | 4 people | 11 (12.5%) | 1 (3.1%) |  |
|  | 5 people | 3 (3.4%) | 3 (9.4%) |  |
|  | More than 5 people | 12 (13.6%) | 5 (15.6%) |  |
| **Perceived adequacy of daily expenses** |  |  |  | .697 |
|  | Very insufficient | 1 (1.1%) | 0 (0.0%) |  |
|  | Not enough | 6 (6.8%) | 1 (3.1%) |  |
|  | Just enough | 63 (71.6%) | 21 (65.6%) |  |
|  | More than enough | 16 (18.2%) | 9 (28.1%) |  |
|  | Very abundant | 2 (2.3%) | 1 (3.1%) |  |
| **Community centre visits per week** |  |  |  | .002 |
|  | 1 time | 4 (4.5%) | 7 (21.9%) |  |
|  | 2 times | 12 (13.6%) | 5 (15.6%) |  |
|  | 3 times | 20 (22.7%) | 4 (12.5%) |  |
|  | 4 times | 15 (17.0%) | 7 (21.9%) |  |
|  | 5 times | 27 (30.7%) | 4 (12.5%) |  |
|  | More than 5 times | 9 (10.2%) | 1 (3.1%) |  |
|  | Others | 1 (1.1%) | 4 (12.5%) |  |
| **Played exergames before** |  |  |  | .039 |
|  | Yes | 47 (53.4%) | 10 (31.2%) |  |
|  | No | 41 (46.6%) | 22 (68.8%) |  |
| **Months of prior exergame experience** |  | 7.15 (6.80) | 5.29 (8.28) | .591 |
|  | Missing | 51 | 25 |  |
| **Participated in I-SING previously** |  |  |  | .036 |
|  | Yes | 29 (33.0%) | 4 (12.5%) |  |
|  | No | 59 (67.0%) | 28 (87.5%) |  |
| **Programme cohort** |  |  |  | 1.000 |
|  | I-SING2024 (4 week) | 0 (0.0%) | 0 (0.0%) |  |
|  | I-SING2024 (8 week) | 22 (75.9%) | 3 (75.0%) |  |
|  | I-SING2023 (6 week) | 0 (0.0%) | 0 (0.0%) |  |
|  | I-SING2023 (12 week) | 0 (0.0%) | 0 (0.0%) |  |
|  | Others | 7 (24.1%) | 1 (25.0%) |  |
|  | Missing | 59 | 28 |  |

## *Note*. Values are mean (SD) or n (%). Percentages are based on nonmissing observations within each completion group. P values are unadjusted. Continuous variables were compared using Welch two-sample t tests; binary categorical variables using Fisher exact tests; and multilevel categorical variables using Monte Carlo permutation Pearson chi-square tests (20,000 replicates).

## Table S4. Internal consistency of the principal multi-item measures at T1

| **Measure** | **Items, n** | **Wave** | **Complete cases, n** | **Cronbach α** |
| --- | --- | --- | --- | --- |
| MHC-SF total | 14 | T1 | 242 | 0.89 |
| Adapted MPAM-R intrinsic motivation score | 14 | T1 | 218 | 0.87 |
| SRHI | 12 | T1 | 201 | 0.9 |

*Note*. Designated missing-value codes and values outside the prespecified response ranges were treated as missing. PASE was not included because its weighted total combines distinct physical activity domains and is not intended to function as a unidimensional multi-item scale. MHC-SF=Mental Health Continuum–Short Form; MPAM-R=Motives for Physical Activity Measure–Revised; SRHI=Self-Report Habit Index; PASE=Physical Activity Scale for the Elderly.

## Table S5: Paired changes and effect sizes for study outcomes

| **Outcome** | **n pairs** | **Mean at T0** | **SD at T0** | **Mean post** | **SD post** | **Cohen dz** | **95% CI low** | **95% CI high** | **P value** |
| --- | --- | --- | --- | --- | --- | --- | --- | --- | --- |
| Healthy lifestyle (HL) | 240 | 0.800 | 0.783 | 0.733 | 0.789 | -0.079 | -0.205 | 0.048 | 0.223 |
| MHC-SF total | 240 | 55.188 | 9.793 | 57.592 | 8.701 | 0.279 | 0.149 | 0.407 | <.001 |
| Intrinsic motivation | 166 | 5.936 | 1.004 | 6.294 | 0.805 | 0.398 | 0.239 | 0.555 | <.001 |
| Extrinsic motivation | 240 | 5.503 | 1.083 | 5.735 | 1.035 | 0.205 | 0.051 | 0.358 | 0.009 |
| Habit strength | 143 | 3.338 | 1.113 | 3.810 | 0.840 | 0.423 | 0.252 | 0.594 | <.001 |
| Self-efficacy | 239 | 3.428 | 0.594 | 3.370 | 0.636 | -0.076 | -0.203 | 0.051 | 0.239 |
| Health citizenship / control (HCON) | 239 | 4.331 | 0.510 | 4.377 | 0.512 | 0.077 | -0.050 | 0.204 | 0.233 |
| Health climate / supportive accountability (HCSA) | 239 | 3.144 | 1.361 | 3.248 | 1.364 | 0.066 | -0.061 | 0.193 | 0.308 |
| Perceived social support | 239 | 5.312 | 1.295 | 5.421 | 1.264 | 0.074 | -0.053 | 0.200 | 0.257 |
| Social contract / responsibility | 235 | 5.149 | 1.499 | 5.428 | 1.349 | 0.173 | 0.044 | 0.302 | 0.009 |
| Community actor | 238 | 3.265 | 1.450 | 3.492 | 1.433 | 0.127 | 0.000 | 0.255 | 0.051 |
| Collective efficacy | 239 | 5.007 | 0.782 | 4.879 | 0.830 | -0.141 | -0.268 | -0.013 | 0.031 |
| Information seeking | 239 | 3.347 | 0.776 | 3.373 | 0.693 | 0.031 | -0.096 | 0.158 | 0.633 |
| Health literacy | 239 | 3.310 | 0.530 | 3.297 | 0.508 | -0.022 | -0.149 | 0.104 | 0.728 |

## *Note*. Effect sizes were calculated using complete pairs. Positive Cohen dz values indicate higher scores at the later wave relative to T0. MHC-SF scores were harmonised to the original 0-to-5 item metric before total scores were calculated.

## Table S6: Adjusted regression models predicting physical activity at post-training (T1) and follow-up (T2)

| Outcome | Term | Estimate | SE | t value | P value | 95% CI lower | 95% CI upper |
| --- | --- | --- | --- | --- | --- | --- | --- |
| T1 Post-training PASE | (Intercept) | 89.497 | 116.325 | 0.769 | 0.445 | -144.149 | 323.143 |
|  | Baseline PASE | 0.546 | 0.139 | 3.918 | <.001 | 0.266 | 0.826 |
|  | Capability composite | -1.809 | 13.405 | -0.135 | 0.893 | -28.734 | 25.117 |
|  | Opportunity composite | 31.681 | 15.808 | 2.004 | 0.050 | -0.069 | 63.432 |
|  | Motivation composite | 0.949 | 13.295 | 0.071 | 0.943 | -25.754 | 27.652 |
|  | Age | -0.349 | 1.422 | -0.245 | 0.807 | -3.205 | 2.508 |
|  | Gender (female vs male) | -6.180 | 19.092 | -0.324 | 0.748 | -44.528 | 32.168 |
|  | Participation year: I-SING 2024 | 14.580 | 49.263 | 0.296 | 0.768 | -84.368 | 113.529 |
|  | Participation year: other | 25.818 | 55.204 | 0.468 | 0.642 | -85.063 | 136.699 |
| T2 follow-up PASE | (Intercept) | 271.862 | 94.430 | 2.879 | 0.006 | 81.783 | 461.940 |
|  | Baseline PASE | 0.586 | 0.106 | 5.538 | <.001 | 0.373 | 0.799 |
|  | Capability composite | 10.869 | 10.351 | 1.050 | 0.299 | -9.966 | 31.704 |
|  | Opportunity composite | 20.138 | 12.012 | 1.676 | 0.100 | -4.041 | 44.316 |
|  | Motivation composite | -14.738 | 9.956 | -1.480 | 0.146 | -34.779 | 5.302 |
|  | Age | -2.342 | 1.163 | -2.015 | 0.050 | -4.682 | -0.002 |
|  | Gender (female vs male) | -2.523 | 14.558 | -0.173 | 0.863 | -31.827 | 26.780 |
|  | Participation year: I-SING 2024 | -43.882 | 36.457 | -1.204 | 0.235 | -117.266 | 29.501 |
|  | Participation year: other | -48.827 | 40.772 | -1.198 | 0.237 | -130.897 | 33.244 |

## Table S7: Summary of T3 game feedback and implications for programme delivery

| **Game / game cluster** | **Overall feedback pattern** | **Common reasons given by participants** | **Delivery / implementation implications** |
| --- | --- | --- | --- |
| Apple Frenzy | Most consistently liked | Easy to play; relaxing; exciting; colourful; easy to score; suitable for older adults | Well suited as a core or introductory game. May help build confidence and early engagement because of its accessible pace and clear task demands. |
| Fishing Frenzy | Frequently liked | Fun; involves “a lot of movements”; helps with exercise; active and engaging | Retain as a key programme game. Particularly relevant for linking enjoyment with functional exercise benefits. Can be highlighted in relation to repeated sit-to-stand practice and lower-limb function. |
| Punch Punch | Commonly liked, but occasionally fatiguing | Exciting; energetic; enjoyable challenge; some participants reported arm soreness or fatigue | Useful for participants who enjoy more vigorous upper-limb movement, but may require pacing, progression, and rest options for those with lower endurance. |
| Whack-a-Mole (1P/2P) | Commonly liked; moderately challenging | Interesting; requires focus and reflexes; enjoyable for those who like challenge | Appropriate as a challenge-based game, particularly for participants who enjoy fast responses. May benefit from graded difficulty or additional familiarisation for less confident players. |
| Chinatown Rush / Red Light Rat Race | More polarising | Some participants found them stimulating, exciting, and mentally engaging; others found them too fast, confusing, or stressful | Best used selectively or after familiarisation. May be more suitable for participants who prefer greater cognitive challenge or faster-paced play. |
| Cat Stack | Frequently least liked | Difficult to balance; frustrating; hard to control; repeated failure reduced enjoyment | Use sparingly or provide additional instruction and practice. May not be ideal as a core game for broad community delivery if frustration outweighs enjoyment. |
| Don’t Forget to Wipe | Frequently least liked | Tiring; not enjoyable; difficult to perform; occasionally linked to poor responsiveness | Consider revising calibration, instructions, or frequency of use. This game may require adaptation if older adults find the movement pattern overly effortful or unrewarding. |
| Ice Breaker | Frequently least liked | Hard to hit targets accurately; tiring; difficult to understand or execute | Use selectively and with clearer demonstration. May need improved calibration or more scaffolding before independent play. |
| Cross-cutting feedback on delivery | Strongly recurrent across open-ended comments | Desire for more game variety; longer play time; clearer instructions; improved tracking; better space; more reminders and support | Future delivery should preserve not only enjoyable game content but also practical supports for access and continuation, especially during free-play or lower-supervision phases. |
